# Developing Effective Radio Frequency Vacuum Drying Processes for Moutan Cortex: Effect on Moisture Migration, Drying Kinetics, Physicochemical Quality, and Microstructure

**DOI:** 10.3390/foods13142294

**Published:** 2024-07-21

**Authors:** Zepeng Zang, Fangxin Wan, Haiwen Jia, Guojun Ma, Yanrui Xu, Qiaozhu Zhao, Bowen Wu, Hongyang Lu, Xiaopeng Huang

**Affiliations:** College of Mechanical and Electrical Engineering, Gansu Agricultural University, Lanzhou 730070, China; zangzp@st.gsau.edu.cn (Z.Z.); wanfx@gsau.edu.cn (F.W.); jiahw@st.gsau.edu.cn (H.J.); magj@gsau.edu.cn (G.M.);

**Keywords:** Moutan Cortex, radio frequency vacuum, drying kinetics, physicochemical quality, microstructure

## Abstract

This study aims to maximize the post-harvest quality of Moutan Cortex and reduce energy consumption. Radio frequency vacuum (RFV) technology was used to dehydrate Moutan Cortex in this study to investigate the effects of different drying temperatures, plate spacing, and vacuum degree on the drying kinetics, physicochemical quality, and microstructure of Moutan Cortex. The results showed that RFV drying shortened the dehydration time of the Moutan Cortex by 10.71–28.57% and increased the drying rate by 15.79–54.39% compared to hot-air drying. The best color (∆E = 6.08 ± 0.28, BI = 26.97 ± 0.98) and relatively high retention of polysaccharides, total phenolics, total flavonoids, antioxidant properties, paeonol, gallic acid, paeoniflorin, and benzoylpaeoniflorin contents were observed in the dried products of Moutan Cortex at a drying temperature of 50 °C, spacing of 90 mm, and vacuum of 0.025 MPa. Analyzing the microstructure, it was found that RFV drying could effectively inhibit the shrinkage and collapse of the cellular structure, and a regular and loose honeycomb pore structure appeared inside the samples, which contributed to the rapid migration of the internal moisture. This study can provide a theoretical reference basis for the selection and application of industrialized processing methods of high-quality Moutan Cortex.

## 1. Introduction

Moutan Cortex is the dried root bark of *Paeonia suffruticosa* Andr, which contains active ingredients such as paeonol, paeoniflorin, and various phenolic compounds [1,2]. It has a variety of functions such as anti-inflammatory, antioxidant, and antibacterial properties, clearing heat and activating blood circulation and removing blood stasis [3,4]. Due to the high moisture content of fresh Moutan Cortex (approx. 50~70% in wet basis) and the mechanical damage often incurred during harvesting, it is often susceptible to environmental pollution and microbial damage during storage and transportation, causing adverse effects such as deterioration, decay, a loss of components, and germ breeding, resulting in reduced nutritional and economic value [5,6]. Furthermore, it can lead to the degradation of some key flavor components and colors, affecting the taste and flavor of the product. This hinders the exploitation of the potential value of agricultural products and the development of industrialized processing. Therefore, exploring scientifically appropriate post-harvest drying techniques for Moutan Cortex to improve drying quality and efficiency, while ensuring its medicinal value and economic benefits, is of great importance for promoting the development of the Moutan Cortex industry.

Drying is a critical step in agricultural product processing, which directly affects the quality, storage, and subsequent use of materials [7,8]. Nevertheless, drying is also a process known to degrade Moutan Cortex quality, especially paeonol, gallic acid, paeoniflorin, and benzoylpaeoniflorin. Currently, traditional dehydration methods such as natural drying, shade drying, and hot-air drying are commonly used for Moutan Cortex. Among these methods, natural drying has the advantages of being cost-effective, being simple to operate, and requiring no additional equipment or energy. However, it is greatly influenced by weather conditions, leading to unstable product quality [9]. Shade drying is a dehydration technique conducted at lower temperatures, which can minimize the degradation of heat-sensitive components. However, it has the disadvantages of a slow drying speed and high space requirements [10]. Due to its good controllability and universality for various materials, hot-air drying has become the most commonly used drying technique for agricultural products. However, this method also has certain limitations, such as high energy consumption, low energy utilization, and suboptimal product quality [11,12]. Therefore, the above drying technology can no longer meet the demand of high-quality development of modern agricultural products. Based on consumers’ pursuit of high-quality food, new drying methods such as microwave vacuum, far-infrared vacuum, spray drying, and freeze drying are gradually being applied to the post-harvest dehydration of agricultural products [13,14]. But each method has its unique advantages and limitations.

Radio frequency vacuum (RFV) drying is a dehydration technique for agricultural products that achieves overall heating through the oscillation of charged ions and the rotation of polar molecules, resulting in frictional heat generation [15,16]. Compared with traditional drying methods, RFV technology has the advantages of high energy efficiency, volumetric heating, and environmental friendliness, etc. [17,18]. It has a small temperature gradient during the heating process, which can ensure a uniform heating rate and thermal effect [19]. Secondly, the vacuum environment can not only decrease the boiling point of water so that the water can be evaporated at a lower temperature, thus improving the drying efficiency, but also reduce the presence of oxygen to prevent the oxidative degradation of the material, which is conducive to the retention of heat-sensitive components in the sample [20,21]. In recent years, RFV drying technology has been applied to the dehydration of various agricultural products [22]. For example, Li et al. [23] investigated the effects of radio frequency drying on the moisture status, dielectric properties, and quality of tiger nuts, and the results demonstrated that the application of RFV significantly improved drying efficiency and accelerated the rate of moisture migration. Mao et al. [24] developed an efficient dehydration process for in-shell walnuts using RFV, which found that RFV drying effectively reduced the oxidative degradation of bioactive substances and the loss of heat-sensitive components. Wang et al. [25] found that RFV drying enhanced heat and mass transfer rates and better preserved bioactive compounds compared to hot-air drying. Therefore, the aforementioned findings indicate that applying RFV technology is both feasible and advantageous to the drying of Moutan Cortex. Compared with the results of microwave vacuum drying of Moutan Cortex by Shang et al. [26] RFV drying could better retain the total phenolic and total flavonoid contents of Moutan Cortex. However, recent studies have shown that RFV drying has certain requirements on the electrical conductivity and dielectric properties of materials and is not applicable to all types of materials, especially certain materials with low dielectric constants. Additionally, although RFV technology has better heating uniformity, in some cases, localized overheating or uneven drying may still occur, which may affect the quality and appearance of the products. Therefore, the future development trend of RFV drying may be shifted from a single RFV-drying strategy to a multi-stage mixed-drying strategy of RFV combined with other dehydration technologies.

To date, current research has primarily focused on the effects of traditional drying methods on the chemical constituents of Moutan Cortex [27]. Chen et al. [28] analyzed the drying kinetics of Moutan Cortex by determining the water content of the dry base during the drying process and interpreted its drying characteristics, which was of some significance in elucidating the drying mechanism of Moutan Cortex. Nevertheless, the study did not determine the physicochemical quality of the samples, and the evaluation index was single. There have been no reports on the effects of RFV drying technology on the dehydration treatment of Moutan Cortex, as well as its influence on drying kinetics, physicochemical properties, and microstructure. In this context, this study comprehensively evaluated the effects of RFV drying on the drying kinetics and physicochemical quality of Moutan Cortex by taking drying characteristics, color, total phenolics, total flavonoids, polysaccharides, antioxidant capacity, microstructures, and natural activity ingredients as evaluation indexes. The aim was to explore a reasonable drying method and process that maximally preserved the original sensory qualities, flavor, and color of Moutan Cortex, with the goal of providing theoretical and data support for high-quality and high-efficiency dehydration processing of Moutan Cortex.

## 2. Materials and Methods

### 2.1. Experimental Materials

Fresh Moutan Cortex was purchased from Zhangxian County, Dingxi City, Gansu Province, and then immediately placed in the refrigerator at 2–4 °C for refrigeration. The uniform length and complete shape of peony peels were selected as test materials, and the initial moisture content (*M*_0_) of the Moutan Cortex was measured as 54 ± 1.12% according to the method of Huang et al. [29].

### 2.2. Experimental Methods

**Figure 1:**
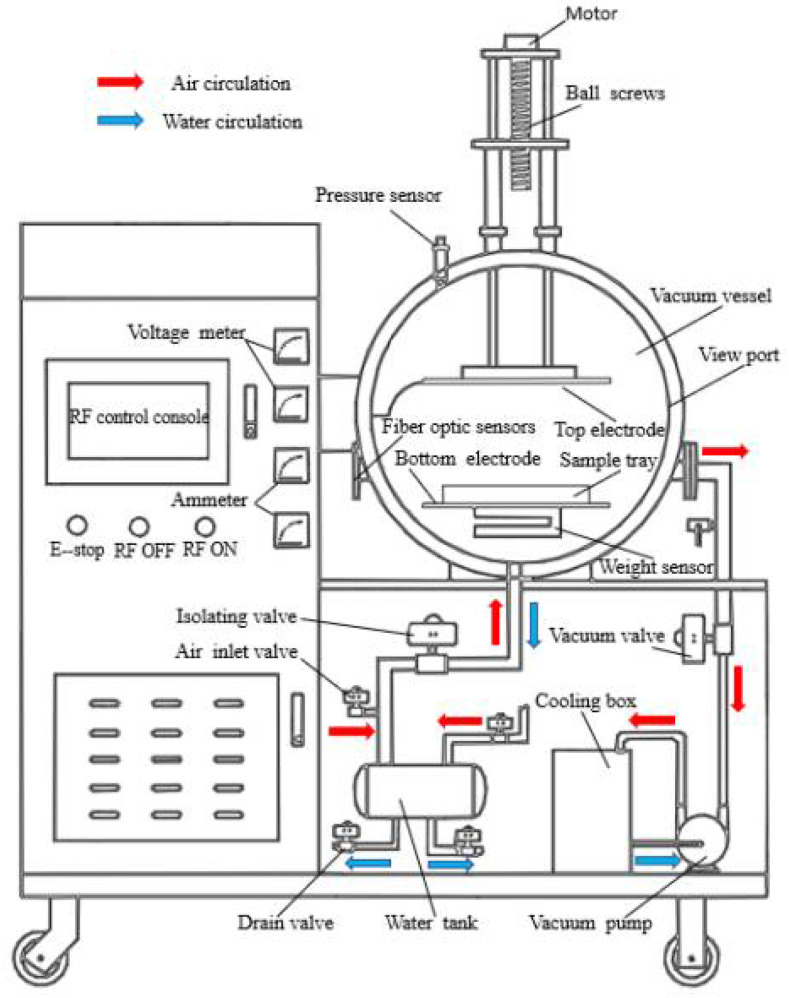
Radio frequency vacuum-drying equipment drawing.

Based on the preliminary pre-experiments of the group, the drying temperature (45 °C, 50 °C, 55 °C), the plate spacing (80 mm, 90 mm, 100 mm), and the vacuum degree (0.015 MPa, 0.025 MPa, 0.035 MPa) were used as the experimental factors for the drying test for Moutan Cortex [30,31]. During the experiment, 200 ± 0.5 g of samples were weighed, evenly spread in a polypropylene porous drying tray (590 mm × 385 mm × 45 mm), and then put into a RFV drying equipment for drying (Figure 1). The experiment was stopped when the wet basis moisture content of the material decreased to 13% [32].

**Figure 2:**
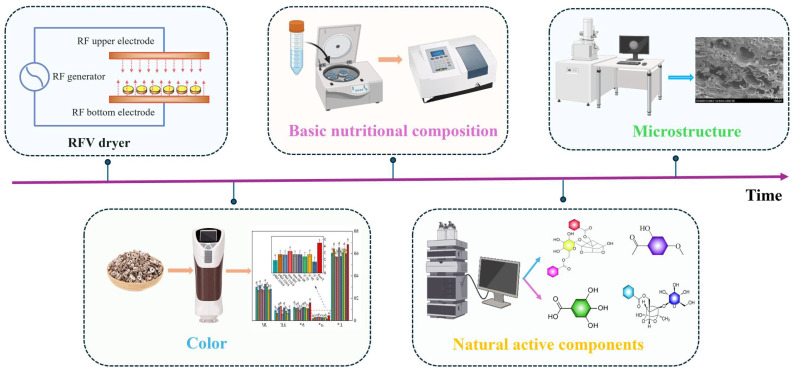
Experimental flow chart of RFV treatment of Moutan Cortex.

Hot-air drying (Control): The selected Moutan Cortex was washed and drained, then precisely weighed to 200 ± 0.5 g. The samples were evenly spread in a single layer on a stainless-steel tray. According to the previous experiments, the parameters of the hot-air drying (GZX-DH400-BS, PingXuan Scientific Instrument Co., Ltd. Shanghai, China) oven were adjusted to the following: temperature 50 °C, wind speed 1.2 m/s, and preheating 30 min. The weight of the Moutan Cortex was recorded every 10 min until the wet basis moisture content decreased to 13%, at which point the experiment was stopped. To ensure the reliability of the experiment, all experiments were measured three times. The experimental flow chart for this study is shown in Figure 2.

### 2.3. Moisture Content and Drying Rate

The moisture ratio (*MR*) and drying rate (*DR*) of the Moutan Cortex was computed using Equations (1) and (2) [33,34].

(1) $$MR=(M_{t}-M_{e})/(M_{0}-M_{e})$$

(2) $$DR=(M_{t}-M_{t-∆t})/∆t$$

where *M_t_* is the dry base moisture content at the drying time *t*, g/g; *M_e_* is the equilibrium water content, g/g; and *M*_0_ is the initial dry basis moisture content, g/g.

### 2.4. Color Measurement

After the dried Moutan Cortex samples were crushed to powder form, the color changes in the dried Moutan Cortex products under different drying conditions were measured using a precision colorimeter [35] (CR-10 colorimeter, Konica Minolta Co., Ltd., Tokyo, Japan). The total color difference (∆*E*) and browning index (*BI*) were calculated according to Equations (3) and (4).

(3) $$∆E=\sqrt{{\left(L^{*}-L_{0}\right)}^{2}+{\;(a^{*}-{\;a}_{0})}^{2}+{\left(b^{*}-{\;b}_{0}\right)}^{2}}$$

(4) BI=(x−0.3/0.17)×100

(5) $$x=(a^{*}+1.75L^{*})/(5.645L^{*}+a^{*}-3.012b^{*})$$

where L*, a*, and b* represent the brightness/darkness, redness/greenness, yellowness/blueness of dried Moutan Cortex, whereas *L*_0_, *a*_0_, and *b*_0_ represent the same for fresh Moutan Cortex.

### 2.5. Determination of Natural Active Substances

The extract in Section 2.6.1 was ultrasonicated and filtered through a 0.22 μm microporous filter membrane to obtain the extract used for the determination of the natural active substances of Moutan Cortex. Then, 1 mg of the control of paeonol, gallic acid, paeoniflorin, and benzoylpaeoniflorin (purity 98%, Chengdu Reflex Biotechnology Co., Ltd., Chengdu, China) was weighed precisely and made into a mixed control solution with the concentrations of 250 μg·mL^−1^, 125 μg·mL^−1^, 62.5 μg·mL^−1^, 31.25 μg·mL^−1^, 15.62 μg·mL^−1^, and 7.81 μg·mL^−1^ using 70% ethanol as the solvent. The main natural active substances were determined by high performance liquid chromatography (HPLC). The conditions were as follows: Agilent Eclipes XDB-C_18_ (250 mm × 4.6 mm, 5 μm), mobile phase: phosphoric acid (B)-1% aqueous solution (D), flow rate of 1.0 mL·min^−1^, column temperature of 40 °C, detection wavelength of 250 nm, injection volume of 1 μL.

### 2.6. Measurement of Basic Nutrient Compound

#### 2.6.1. Preparation of Polysaccharides, Total Phenolics, Total Flavonoids, and Antioxidant Extracts

After drying, the Moutan Cortex samples were ground and passed through an 80-mesh sieve. And 0.5 g was weighed precisely, and 20 mL of 75% ethanol was added into a 50 mL centrifuge tube, which was rotated and shaken in a shaker (250 r/min) under dark conditions at room temperature for 48 h. Then, the mixture was centrifuged for 15 min (4 °C, 3000 r/min). Finally, the supernatant was collected and diluted to 25 mL with 75% ethanol. The extract was stored in a constant temperature refrigerator at 4 °C for the determination of polysaccharides, total phenols, total flavonoids, and antioxidant content.

#### 2.6.2. Determination of Polysaccharide Content

The polysaccharide content was determined by the sulfuric acid–phenol method [36], which was calibrated with sucrose as the standard, and the standard curve was obtained as *Y* = 0.0084*X* − 0.1634 (*R*^2^ = 0.9936). The polysaccharide content was calculated using Formula (6).

(6) $$P_{c}=(V_{T}\times C_{1})/(V_{M}\times M)$$

where *P_c_* is the polysaccharide content, mg/g; *V_T_* is the total volume of the sample extract, mL; *C*_1_ is sucrose mass concentration; *V_M_* is the volume of sample extract used in the titration, mL; and *M* is the mass of dry matter of Moutan Cortex, g.

#### 2.6.3. Determination of Total Phenolic Content

The total phenolic content was determined by the Folin–Ciocalteu reagent method [37], which was calibrated with gallic acid as standard, and the standard curve was obtained as *Y* = 0.0036X + 0.0326 (*R*^2^ = 0.9972). The total phenolic content was calculated using Formula (7).

(7) $$TPC=\;(V_{T}\times C_{2})/(V_{M}\times M)$$

where *TPC* is the total phenol content, mg/g, and *C*_2_ is the content of gallic acid in the sample assay tube obtained from the standard curve.

#### 2.6.4. Determination of Total Flavonoid Content

The total flavonoid compounds were determined by the sodium NaNO_2_-Al(NO_2_)_3_-NaOH method [38]. The standard curve equation of total flavonoids was obtained as Y = 0.0053*X* + 0.0037 (*R*^2^ = 0.9984) using catechin as a standard for calibration. The total flavonoid content was calculated as follows:

(8) $$TFC=(V_{T}\times C_{3})/(V_{M}\times M)$$

where *TFC* is the total flavonoid content, mg/g; *C*_3_ is the mass concentration of catechin, mg/mL.

#### 2.6.5. Determination of Antioxidant Activity

The antioxidant activities in Moutan Cortex extracts were measured using the DPPH free-radical scavenging activity, according to the method of Zang et al. [11] and Xu et al. [39].

### 2.7. Microstructure

The surface microstructure of Moutan Cortex was observed using a modified version of the method by Wang et al. [40]. The samples were cut into 5 mm × 5 mm slices and coated with an ion sputtering coater for 90 s to prepare them for microstructural observation. The accelerating voltage was set to 5 kV, and the magnification was 300×. Representative fields of view were selected for photographing.

### 2.8. Statistical Analysis

Origin 8.5 software and Microsoft Excel 2010 were used to plot and analyze the quality and drying data. The experimental data were analyzed by SPSS Statistics 22.0 software, and the differences between samples were analyzed by one-way analysis of variance (ANOVA). To ensure the reliability of the experiment, all experiments were measured three times.

## 3. Results and Discussion

### 3.1. Drying Characteristics

#### 3.1.1. Effect of Drying Temperatures on the Drying Characteristics

**Figure 3:**
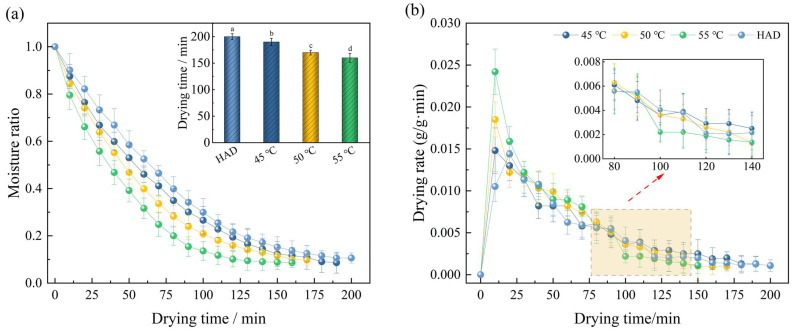
Effects of drying temperature on drying curve (**a**) and drying rate curve (**b**). Note: Values with different letters represent significant difference at the level of *p* < 0.05.

The effects of different drying temperatures on the moisture ratio and drying rate of Moutan Cortex after RFV drying when the plate spacing was 90 mm and the vacuum degree was 0.025 MPa are shown in Figure 3. The corresponding drying times after treatment at 45 °C, 50 °C, and 55 °C were 190 min, 170 min, and 160 min, respectively, which reduced the drying time by 5.0%, 15.0%, and 20.0%, respectively, compared with that of hot-air drying. The average drying rates at the corresponding pretreatment times were 0.55 g/g·min, 0.62 g/g·min, and 0.73 g/g·min, which were 7.84%, 21.57%, and 43.14% higher than those of the control, respectively. This indicates that RFV drying can effectively reduce the internal diffusion resistance and energy consumption in the dehydration process of Moutan Cortex [41]. Compared with 4 °C , the drying time at 50 °C and 55 °C was shortened by 12.73% and 32.73%, which may be because with the increase in temperature, the temperature gradient, pressure gradient, and solute concentration gradient between the drying medium and the different organizational structures of the Moutan Cortex increased, which to a certain extent destroyed the organizational structure of the material, enhanced the evaporation and diffusion of internal water, and improved the heat and mass transfer rate [18,42]. Therefore, the drying time was shortened, and the drying rate was increased.

#### 3.1.2. Effect of Plate Spacing on Drying Characteristics

**Figure 4:**
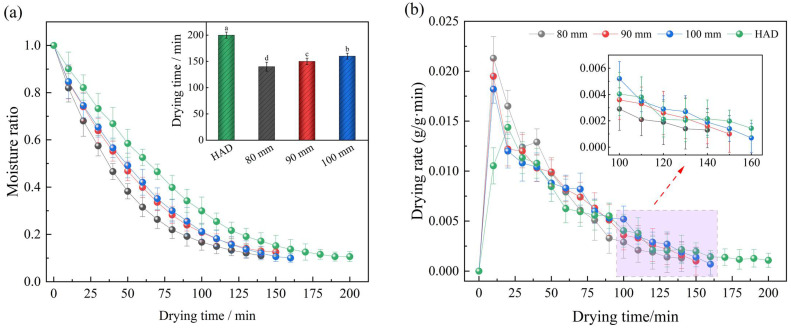
Effects of plate spacing on drying curve (**a**) and drying rate curve (**b**). Note: Values with different letters represent significant difference at the level of *p* < 0.05.

The effects of different plate spacing on the moisture ratio and drying rate of Moutan Cortex after RFV drying when the drying temperature was 50 °C and the vacuum degree was 0.025 MPa are shown in Figure 4. The time to dry the samples to a safe moisture content was 140 min, 150 min, and 160 min for plate spacings of 80 mm, 90 mm, and 100 mm, respectively, which reduced the dehydration time by 30.0%, 25.0%, and 20.0%, respectively, when compared to hot-air drying (200 min). This indicates that the reduction in the pole plate spacing had a positive effect on shortening the drying time and improving the drying rate [43]. At constant initial moisture content, the RF electric field strength increased with decreasing plate spacing, allowing more energy to be absorbed by the sample. Secondly, when lowering the plate spacing, the capacitance between the plates increased, causing a decrease in the frequency of the heating circuit and, therefore, an increase in the drying rate [44]. However, too low a spacing between the poles could caused the frequency of the heating circuit to be lower than the frequency of the generator circuit, resulting in a decrease in the degree of coupling between the two and an uncontrolled heating of the corners or edges of the sample, which resulted in a weakening of the energy transfer and a decrease in the drying quality [21]. When the plate spacing was too large, due to the different dielectric properties of the material and the surrounding medium, it could lead to the uneven distribution of the electric field, thus causing the phenomenon of uneven heating on the surface and edge of the sample, which reduced the physicochemical quality and medicinal value of the Moutan Cortex. Therefore, to ensure good heating uniformity and an acceptable heating rate, the suitable plate spacing for RFV drying of Moutan Cortex was 90 mm.

#### 3.1.3. Effect of Vacuum Degree on Drying Characteristics

**Figure 5:**
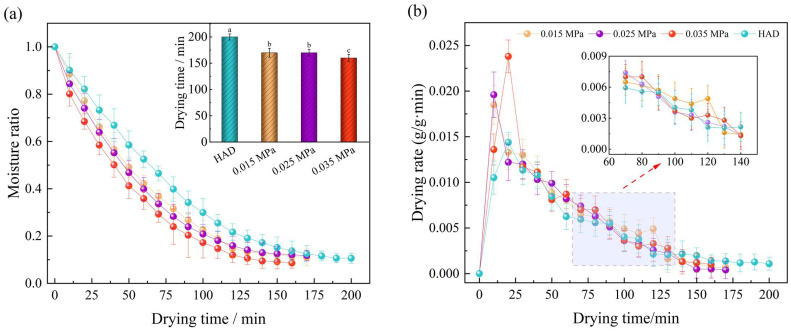
Effects of vacuum degree on drying curve (**a**) and drying rate curve (**b**). Note: Values with different letters represent significant difference at the level of *p* < 0.05.

The effects of different vacuum degrees on the moisture ratio and drying rate of Moutan Cortex are shown in Figure 5. With the increase in vacuum degree, the dehydration time of the material was shortened, and the drying rate increased. At a vacuum degree of 0.035 MPa, the total drying time was 160 min, which was 5.88% lower than the drying time at vacuum degree of 0.015 MPa (170 min) and 0.025 MPa (170 min), respectively. The average drying rates at the corresponding vacuum levels were 0.65 g/g∙min and 0.62 g/g∙min, representing reductions of 7.14% and 11.43%, respectively, compared to that at a vacuum degree of 0.035 MPa (0.70 g/g∙min). On the one hand, because of the increase in vacuum degree, the total pressure difference between the inside and outside of the sample increased. On the other hand, the increased in vacuum degree can lead to a decrease in the boiling point of water, which is not only conducive to the removal of water from the Moutan Cortex but also prevents oxidative damage of heat-sensitive substances [45]. Furthermore, it was found that the use of a lower vacuum degree would lead to frequent glow discharge phenomena in the drying process, making the surface of the material locally overheated and even scorched. It was worth noting that the dehydration time and drying rate of the samples were basically similar under the conditions of a vacuum degree of 0.015 MPa and 0.025 MPa, and there was no significant difference between them (*p* > 0.05).

### 3.2. Color

**Figure 6:**
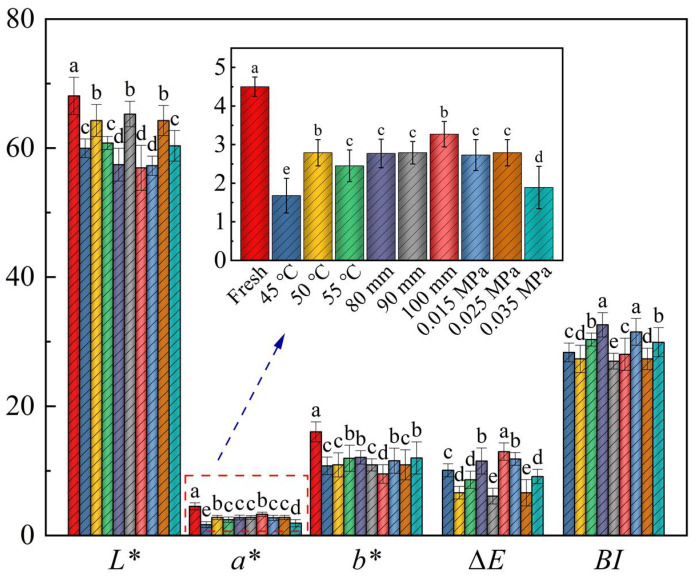
Color of Moutan Cortex subjected to various drying conditions. Note: Values with different letters represent significant difference at the level of *p* < 0.05.

Color is one of the most important indicators for assessing the quality, nutritional value and taste of food products, and is important for product appearance quality, economic value, and consumer experience [46,47]. Enzymatic and non-enzymatic browning, pigment degradation, Maillard reaction, and ascorbic acid oxidation occurring during the drying process contribute to the deterioration of sample color quality, thereby leading to a decline in product quality [48]. Fresh samples of Moutan Cortex were used as the control to compare the total color difference (∆*E*) and L*, a*, and b* of the dried products under different drying conditions (Figure 6). It was found that there were significant differences in the effects of different drying conditions on the color attributes of Moutan Cortex (*p* < 0.05). The L*, a*, and b* of the samples were reduced after RFV drying compared to the fresh samples. As the drying temperature increased, the color difference of the samples initially decreased and then increased. At 50 °C, the lightness (L*) was highest (64.26 ± 2.34), and the color difference (∆*E*) was the lowest (6.61 ± 0.45). This indicated that appropriately increasing the temperature can improve the color of the samples [49]. This may be due to the increased probability of oxidative degradation of heat-sensitive compounds within the Moutan Cortex when it is in contact with hot and humid air for a long period of time at lower drying temperatures, leading to a significant decrease in the brightness value. However, with the increase in temperature, the phenols, glycosides, anthocyanosides, as well as other substances in the sample, were more sensitive to light and heat and were prone to degradation and producing brown polymers [50,51], as well as exacerbating the Maillard reaction, which made the L* value lower and browning more serious. The effect of vacuum degree and plate spacing on the color of Moutan Cortex was similar to those of drying temperature.

### 3.3. Natural Active Components Content

**Figure 7:**
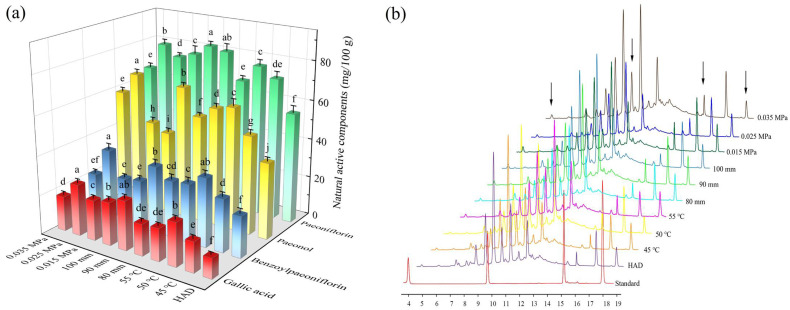
Natural active component content (**a**) and chromatographs (**b**) of Moutan Cortex subjected to various drying conditions. Note: Values with different letters represent significant difference at the level of *p* < 0.05.

Paeonol, gallic acid, paeoniflorin, and benzoylpaeoniflorin, as the main natural active ingredients in Moutan Cortex, have antioxidant, anti-atherosclerotic, blocking cellular degeneration, and enhancing immunomodulation effects. Figure 7 illustrates the impact of different drying conditions on the natural bioactive components in Moutan Cortex. Compared to hot-air drying, the retention of paeonol, gallic acid, paeoniflorin, and benzoylpaeoniflorin significantly increased after RFV drying. This indicated that the thermal and oxidative damage to the natural active substances during RFV drying was small, and therefore, the loss of natural active compounds was effectively reduced. Analyzing the effects of different drying conditions on their contents, it was found that the contents of paeonol, gallic acid, paeoniflorin, and benzoylpaeoniflorin showed a tendency to increase and then decrease with the increase in drying temperature, and the contents were higher at the drying temperature of 50 °C, which were 62.28 mg/100 g, 22.16 mg/100 g, 76.83 mg/100 g, and 34.98 mg/100 g, respectively. This may be because the active ingredients in Moutan Cortex are mostly heat-sensitive substances, which are easy to decompose at too-high temperatures, which is not conducive to the retention of the content. At a drying temperature of 50 °C and a vacuum degree of 0.025 MPa, it was found that when the electrode distance was 90 mm, the contents of paeonol and paeoniflorin reached their maximum values, at 67.28 mg/100 g and 82.83 mg/100 g, respectively. In addition, the drying conditions had the greatest influence on the retention of paeonol content, especially the vacuum degree. It was found that the highest paeonol content was 0.025 MPa (69.28 mg/100 g), which increased the retention rate by 79.06%, 51.73%, and 18.69% compared with HAD, 0.015 MPa, and 0.025 MPa. This may be because smaller electrode spacing results in a high-intensity electromagnetic field, which can easily cause cellular damage and lead to the degradation of paeonol and paeoniflorin. Therefore, an appropriate increase in the spacing of the plates was favorable for the retention of the natural active substances. Analyzing the effects of different vacuum levels on the active ingredients, it was found that the highest content of paeoniflorin and benzoylpaeoniflorin was obtained at a vacuum level of 0.025 MPa. This is because suitable vacuum conditions not only reduce the oxygen level and inhibit the activity of enzymes that degrade the active compounds but also better maintain the structural integrity of the plant material, which helps to retain the natural active compounds embedded in the cellular structure.

### 3.4. Polysaccharides Content

**Figure 8:**
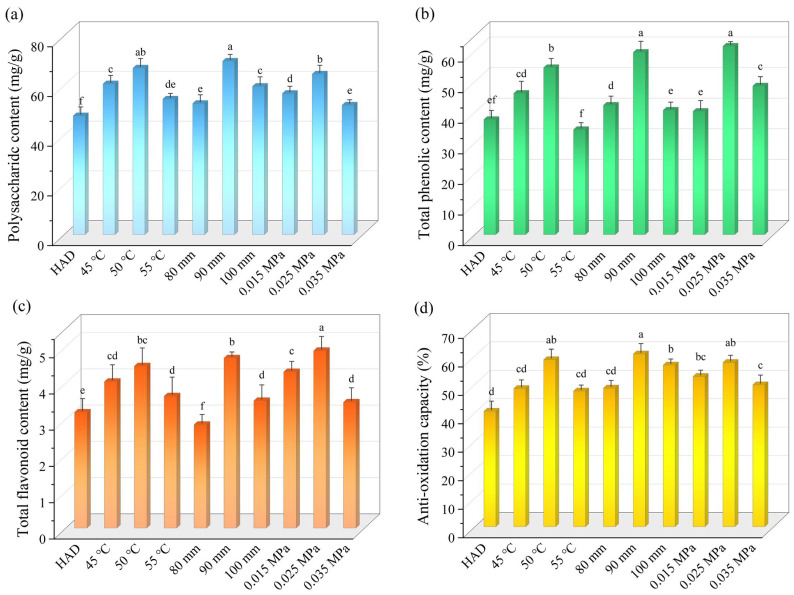
Effects of different drying conditions on polysaccharides (**a**), total phenolics (**b**), total flavonoids (**c**), and anti-oxidant activity (**d**) of Moutan Cortex. Note: Values with different letters represent significant difference at the level of *p* < 0.05.

The effects of different drying conditions on the polysaccharide content of Moutan Cortex are shown in Figure 8a. The polysaccharide content of Moutan Cortex after hot-air drying was 47.79 ± 2.89 mg/g. The retention of polysaccharide content increased significantly after RFV drying. This is because high temperatures during hot-air drying destroy the molecular structure of polysaccharides, leading to their thermal degradation into smaller sugar molecules, thus reducing the total polysaccharide content. Additionally, contact with oxygen in the air can easily trigger oxidation reactions, further reducing the polysaccharide content [52]. With the increase in drying temperature, the polysaccharide content in the samples initially increased and then decreased. At a temperature of 50 °C, the polysaccharide content was 67.01 ± 3.01 mg/g, which was 9.48% and 18.59% higher compared to 45 °C and 55 °C, respectively. This trend may be attributed to the extended drying time at 45 °C, which can lead to some degradation of polysaccharides. At 55 °C, the higher temperature resulted in localized overheating and scorching, exacerbating the Maillard reaction between reducing sugars and amino acids, thus leading to a decrease in polysaccharide content [53]. Furthermore, the high temperature caused protein denaturation and cell wall decomposition in the food matrix, while the intermolecular movement was intense, resulting in the breakage of the sugar chain. The polysaccharide content was 52.76 ± 2.89 mg/g, 59.79 ± 2.23 mg/g, and 59.64 ± 3.22 mg/g at 80 mm, 90 mm, and 100 mm plate spacing, respectively. This is because a greater distance between the electrodes reduces the frequency of collisions among polar molecules within the material during the drying process [21], thereby preserving the integrity and content of polysaccharides in the Moutan Cortex. The effect of vacuum degree on the polysaccharide content of Moutan Cortex was similar to that of temperature and plate spacing.

### 3.5. Total Phenolic Content

Phenolic compounds are a class of thermosensitive substances with antioxidant properties [54,55]. The effects of different drying conditions on the total phenolic content of Moutan Cortex are shown in Figure 8b.

The total phenolic content (54.58 ± 1.45 mg/g) of the dried products of Moutan Cortex at a drying temperature of 50 °C was higher than that of other drying temperature conditions and hot-air drying. This indicated that an appropriate drying temperature was conducive to the retention of phenolic compounds. At lower temperatures, the drying time of the samples was longer, leading to the substantial degradation of phenolics under the combined action of polyphenol oxidase and peroxidase. As the temperature increased, the drying time was significantly shortened, the time for the oxidation reaction to occur was correspondingly reduced, and the total phenolic content increased. It is noteworthy that when the temperature exceeds 50 °C, the degradation reaction rate of phenolics was rapidly accelerated, resulting in a decrease in the total phenolic content. Under high-temperature conditions, although the drying time is reduced to some extent, it increases the activity of polyphenol oxidase, which further promotes the oxidative degradation of phenolics. The highest total phenolic content was found in the dried Moutan Cortex products at a vacuum degree of 0.025 MPa. This may be attributed to the increased vacuum enhancing the density and intensity of the electric field distribution in the RFV environment. Additionally, radio frequency can cleave covalently bound polyphenolic compounds from plant tissue cells and disrupt cell walls, thereby releasing phenolic substances. Analyzing the changes in total phenolic content under different plate spacings revealed that a plate spacing of 90 mm caused the least loss of phenolic compounds. This indicates that the temperature of the sample increased rapidly under the effect of “ion migration” and “dipole rotation” loss, and the phenolic compounds were formed by the ester bond with xylan in the cell wall, and the sample absorbed more thermal energy during the heating process, which destroyed the covalent bond of the phenolic compounds and facilitated the solubilization of phenolic substances.

### 3.6. Total Flavonoid Content

Figure 8c reflects the variation rule of total flavonoid content in Moutan Cortex under different drying conditions. As can be seen from the figure, the variation range of total flavonoids content after RFV treatment was 2.85 ± 0.24~4.89 ± 0.35 mg/g. Except for the 80 mm condition, the total flavonoids content of the other drying samples was higher than that of hot-air drying (3.19 ± 0.34 mg/g). Among them, the total flavonoid content showed a tendency of decreasing and then increasing with increasing temperature, which might be attributed to the fact that the increase in temperature led to the enhancement of the activity of some enzymes in the samples, which accelerated their chemical reactions and promoted the metabolism and degradation of the total flavonoids in the Moutan Cortex. Nevertheless, with a proper increase in temperature, the hydrogen bonding structure inside the sample became more stable, and reduction reactions were more likely to occur in the redox processes, thus improving the stability of the flavonoids in Moutan Cortex. Analyzing the impact of vacuum degree on the total flavonoid content in dried Moutan Cortex revealed that under 0.025 MPa, the total flavonoid content increased by 11.86% and 29.04% compared to 0.015 MPa and 0.035 MPa, respectively. Higher vacuum degree causes significant damage to the internal tissue structure of the material, while lower vacuum degrees may prolong drying time, increasing the exposure of flavonoid compounds to heat and oxygen, thus accelerating their degradation. Similar conclusions were found by Xu et al. [21] in the RFV drying of wolfberry (Lycium barbarum). Therefore, optimal vacuum conditions can minimize these effects, maintain higher flavonoid content, and ensure better quality of the dried products.

### 3.7. DPPH Free-Radical Scavenging Capacity

In this study, the antioxidant activity of the samples was measured using the DPPH method, which is based on the ability of antioxidants to scavenge 2,2-diphenyl-1-picrylhydrazyl radicals. The effects of different drying conditions on the antioxidant activity of Moutan Cortex are shown in Figure 8d. The highest free-radical scavenging activity of Moutan Cortex (60.65%) was observed at the radiofrequency drying parameters of a drying temperature of 45 °C, plate spacing of 90 mm, and vacuum degree of 0.025 MPa, which was increased by 49.57% compared to that of hot-air drying (40.55%). This was attributed to the longer time required for hot-air drying and the increased contact time between the oxygen in the air and the active components with antioxidant properties in the material [56], leading to a decrease in the antioxidant capacity. Additionally, analyzing the antioxidant capacity of Moutan Cortex at different drying temperatures, it was found that the free-radical scavenging rates of the samples were 48.55%, 58.71%, and 47.65% when the temperatures were 45 °C, 50 °C, and 55 °C, respectively. This indicates that a proper increase (when the temperature is raised from 45 °C to 50 °C) in temperature is beneficial to improve the antioxidant capacity of Moutan Cortex. However, excessively high temperatures can lead to the oxidation and degradation of antioxidants and natural active substances, thereby weakening their antioxidant capacity to some extent [57]. The plate spacing and vacuum degree show a similar trend. This study found that the variation in the antioxidant capacity of Moutan Cortex does not entirely correspond to changes in total phenolics and total flavonoid content. This discrepancy may be attributed to the fact that the total antioxidant activity was the collective result of various antioxidant components in the sample, and there may be synergistic or antagonistic interactions among different antioxidant components [58]. Thus, this explains the observed inconsistency.

### 3.8. Microstructure

**Figure 9:**
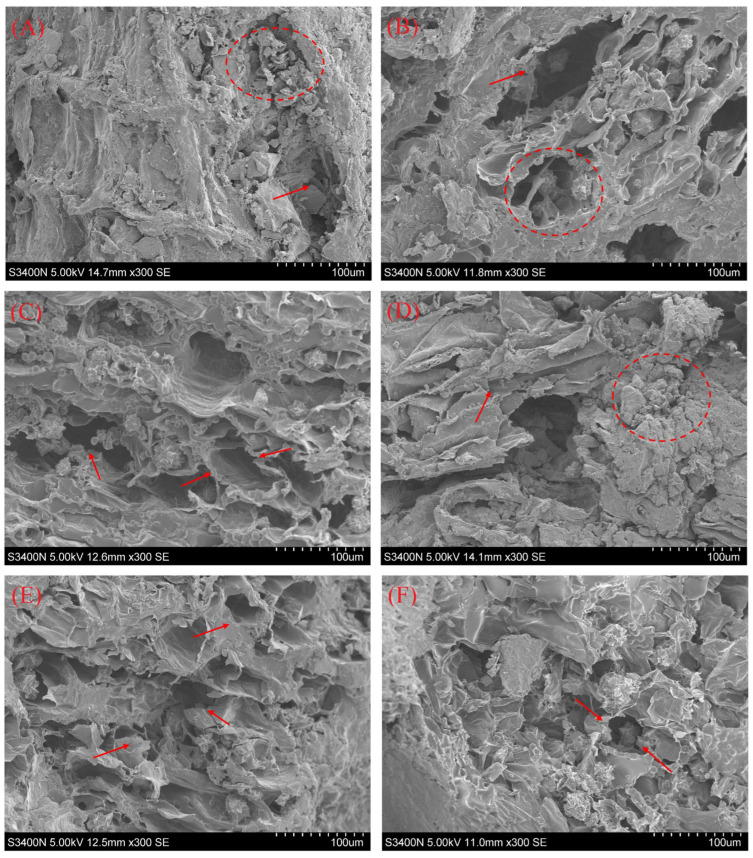
Effects of different drying conditions on the microstructure of Moutan Cortex. (**A**) Hot-air drying, (**B**) 45 °C/90 mm/0.025 MPa, (**C**) 50 °C/90 mm/0.025 MPa, (**D**) 55 °C/90 mm/0.025 MPa, (**E**) 50 °C/90 mm/0.035 MPa, and (**F**) 50 °C/100 mm/0.025 Mpa (Red markers indicate cracked and damaged microstructures; red markers indicate shrunk and deformed microstructures).

The microstructural changes in the material are closely related to the internal moisture diffusion and heat and mass transfer rates during the drying process. The microstructures of samples under different drying conditions are shown in Figure 9. The hot-air drying sample showed a high-density, less-organized, and irregular cell structure, which appeared to be broken and collapsed. This is because prolonged exposure of Moutan Cortex to high-temperature and high-humidity environments can lead to severe damage to cell walls, causing shrinkage and deformation. This damage may disrupt the integrity of tissue cells. Additionally, hot-air drying can cause surface hardening or case hardening, trapping moisture inside and thereby increasing resistance to moisture diffusion and reducing the rate of heat and mass transfer. By contrast, compared to hot-air drying, RFV drying results in the formation of partially regular and ordered pore structures within the Moutan Cortex. Figure 9 shows that in relatively low-temperature environments, the internal tissue cells of Moutan Cortex were affected by temperature and humidity gradient stress, resulting in a denser internal structure with severe deformation and shrinkage. The microporous channels were fewer and highly blocked. At a drying temperature of 55 °C, the sample surface exhibited gelatinization of starch granules, forming a dense barrier layer that adhered to the surface. The cell tissue was damaged, forming large cavities, while the cell wall structure was accompanied by a greater degree of fracture. Better preservation of the internal pore structure of the samples was observed at a drying temperature of 50 °C, plate spacing of 90 mm, and vacuum degree of 0.025 MPa, when the internal tissue structure of the samples was destroyed to a lower degree and the honeycomb pore structure was more obvious. Therefore, the appropriate drying temperature, plate spacing, and vacuum degree can inhibit the contraction of tissue cells and the collapse of the structure, which has a positive effect on accelerating the migration of water inside the Moutan Cortex and reducing solid–liquid mass transfer resistance.

### 3.9. PCA Analysis and Correlation Analysis

**Figure 10:**
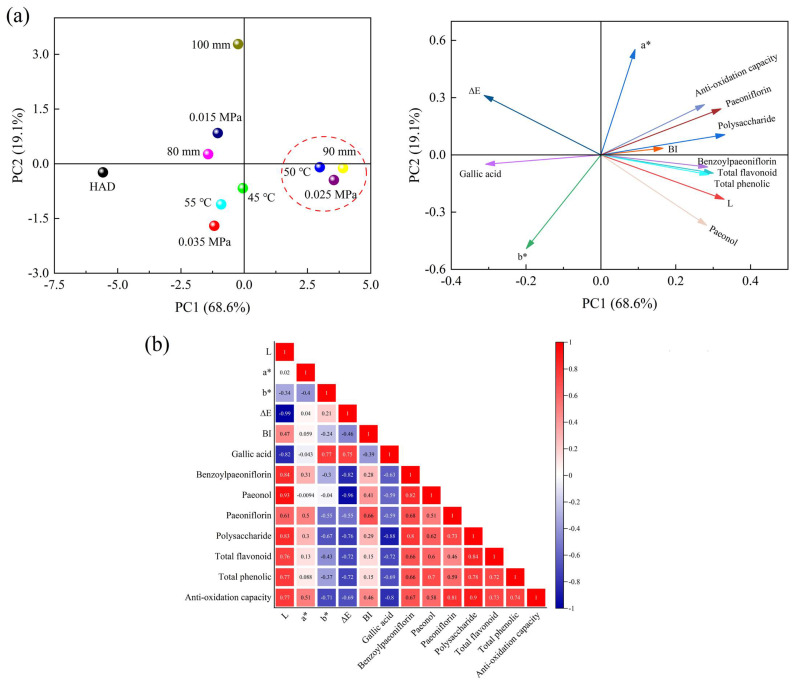
PCA (**a**) and Pearson correlation analysis (**b**) plot of physicochemical quality obtained from different drying treatments.

PCA and correlation analysis were conducted on 13 quality attributes, as shown in Figure 10a. The contribution rates of the first principal component (PC1) and the second principal component (PC2) were 68.6% and 19.1%, respectively, with a total contribution rate of 87.7%, which can explain the total variance in the dataset. In PC1, peonidin and total phenols showed a high positive correlation, while total color difference (∆E) was significantly negatively correlated with brightness (L*), consistent with the conclusions obtained from color analysis. Figure 10b illustrates the PCA among various quality attributes, which provides a better understanding of the relationships between variables, where the darkness of the color indicates the magnitude of the correlation coefficient. This study found a positive correlation between peonidin and benzoyl paeoniflorin, possibly due to their mutual thermosensitive nature and synergistic effects. There was a significant negative relationship between ∆*E* and L* (r = −0.99, *p* < 0.05).

## 4. Conclusions

This paper investigates the effects of RFV drying on the drying characteristics, quality attributes, and microstructure of Moutan Cortex. The results showed that compared with hot-air drying, RFV drying could significantly improve drying efficiency. With the increase in drying temperature and vacuum degree and the decrease in plate spacing, the drying time of the samples was shortened, and the average drying rate was increased. The effects of different drying conditions on the physicochemical properties of Moutan Cortex varied significantly (*p* < 0.05). The highest retention of polysaccharides, total phenolics, total flavonoids, antioxidant properties, paeonol, gallic acid, paeoniflorin, and benzoylpaeoniflorin content was observed in Moutan Cortex at drying temperature of 50 °C, a pole spacing of 90 mm, and a vacuum degree of 0.025 MPa. The findings demonstrated that the optimal drying temperature, plate spacing, and vacuum could better retain the essential nutrients and natural active compounds in the samples. Furthermore, the combined effects of different drying conditions on the quality characteristics were revealed by principal component analysis and Pearson correlation analysis. Overall, as a novel dehydration technology for agricultural products, RFV drying has significant advantages in improving drying efficiency and physicochemical quality of Moutan Cortex.

## Data Availability

The original contributions presented in the study are included in the article, further inquiries can be directed to the corresponding author.

## References

[1] Zou C., Chen Q., Li J., Lin X., Xue X., Cai X., Chen Y., Sun Y., Wang S., Zhang Y. (2024). Identification of potential anti-inflammatory components in Moutan Cortex by bio-affinity ultrafiltration coupled with ultra-performance liquid chromatography mass spectrometry. Front. Pharmacol..

[2] Meng L., Chen Y., Zheng Z., Wang L., Xu Y., Li X., Xiao Z., Tang Z., Wang Z. (2024). Ultrasound-Assisted Extraction of Paeonol from Moutan Cortex: Purification and Component Identification of Extract. Molecules.

[3] Zhang M., Yang L., Zhu M., Yang B., Yang Y., Jia X., Feng L. (2022). Moutan Cortex polysaccharide ameliorates diabetic kidney disease via modulating gut microbiota dynamically in rats. Int. J. Biol. Macromol..

[4] Li C., Li M., Yang H., Li P., Gao W. (2018). Rapid characterization of chemical markers for discrimination of Moutan Cortex and its processed products by direct injection-based mass spectrometry profiling and metabolomic method. Phytomedicine.

[5] Hu D., Yang G., Liu X., Qin Y., Zhang F., Sun Z., Wang X. (2024). Comparison of different drying technologies for coffee pulp tea: Changes in color, taste, bioactive and aroma component. LWT-Food Sci. Technol..

[6] Wu B., Guo X., Guo Y., Ma H., Zhou C. (2021). Enhancing jackfruit infrared drying by combining ultrasound treatments: Effect on drying characteristics, quality properties and microstructure. Food Chem..

[7] Zang Z., Wan F., Ma G., Xu Y., Wang T., Wu B., Huang X. (2024). Enhancing peach slices radio frequency vacuum drying by combining ultrasound and ultra-high pressure as pretreatments: Effect on drying characteristics, physicochemical quality, texture and sensory evaluation. Ultrason. Sonochem..

[8] An K., Fu M., Zhang H., Tang D., Xu Y., Xiao G. (2019). Effect of ethyl oleate pretreatment on blueberry (*Vaccinium corymbosum* L.): Drying kinetics, antioxidant activity, and structure of wax layer. J. Food Sci. Technol..

[9] Jiang C., Wan F., Zang Z., Zhang Q., Ma G., Huang X. (2022). Effect of an Ultrasound Pre-Treatment on the Characteristics and Quality of Far-Infrared Vacuum Drying with Cistanche Slices. Foods.

[10] Tao Y., Han M., Gao Y., Han Y., Show P.L., Liu C., Ye Y., Xie G. (2019). Applications of water blanching, surface contacting ultrasound-assisted air drying, and their combination for dehydration of white cabbage: Drying mechanism, bioactive profile, color and rehydration property. Ultrason. Sonochem..

[11] Zang Z., Zhang Q., Huang X., Jiang C., He C., Wan F. (2023). Effect of Ultrasonic Combined with Vacuum Far-infrared on the Drying Characteristics and Physicochemical Quality of Angelica sinensis. Food Bioprocess Technol..

[12] Shi X., Yang Y., Li Z., Wang X., Liu Y. (2020). Moisture transfer and microstructure change of banana slices during contact ultrasound strengthened far-infrared radiation drying. Innov. Food Sci. Emerg. Technol..

[13] Zhang M., Wu C., Zhang H., Yang N., Wang C., Jike X., Zhang T., Lei H. (2024). Comparison of different drying technologies for kiwifruit pomace: Changes in physical characteristics, nutritional properties and antioxidant capacities. Food Chem..

[14] Yamchi A.A., Sharifian F., Khalife E., Kaveh M. (2024). Drying kinetic, thermodynamic and quality analyses of infrared drying of truffle slices. J. Food Sci..

[15] Zhou X., Wang S. (2019). Recent developments in radio frequency drying of food and agricultural products: A review. Dry. Technol..

[16] Zhou X., Li R., Lyng J.G., Wang S., Wang S. (2018). Dielectric properties of kiwifruit associated with a combined radio frequency vacuum and osmotic drying. J. Food Eng..

[17] Huang Z., Chen L., Wang S. (2015). Computer simulation of radio frequency selective heating of insects in soybeans. Int. J. Heat Mass Transf..

[18] Huang Z., Zhang B., Marra F., Wang S. (2016). Computational modelling of the iMPact of polystyrene containers on radio frequency heating uniformity improvement for dried soybeans. Innov. Food Sci. Emerg. Technol..

[19] Mahmood N., Liu Y., Munir Z., Zhang Y., Niazi B.M.K. (2022). Effects of hot air assisted radio frequency drying on heating uniformity, drying characteristics and quality of paddy. LWT-Food Sci. Technol..

[20] Zang Z., Huang X., He C., Zhang Q., Jiang C., Wan F. (2023). Improving Drying Characteristics and Physicochemical Quality of Angelica sinensis by Novel Tray Rotation Microwave Vacuum Drying. Foods.

[21] Xu Y., Zang Z., Zhang Q., Wang T., Shang J., Huang X., Wan F. (2022). Characteristics and Quality Analysis of Radio Frequency-Hot Air Combined Segmented Drying of Wolfberry (*Lycium barbarum*). Foods.

[22] Shewale S.R., Rajoriya D., Bhavya M.L., Hebbar H.U. (2019). Application of radiofrequency heating and low humidity air for sequential drying of apple slices: Process intensification and quality improvement. LWT-Food Sci. Technol..

[23] Li M., Tian Y., Jiang L., Xu J., Li R., Wang S. (2024). Developing effective radio frequency drying processes for tiger nuts: Dynamic analysis of moisture state, dielectric properties and quality. J. Food Eng..

[24] Mao Y., Wang S. (2021). Simultaneous hot-air assisted radio frequency drying and disinfestation for in-shell walnuts using a two-stage strategy. LWT-Food Sci. Technol..

[25] Wang C., Kou X., Zhou X., Li R., Wang S. (2021). Effects of layer arrangement on heating uniformity and product quality after hot air assisted radio frequency drying of carrot. Innov. Food Sci. Emerg. Technol..

[26] Shang J., Ma G., Wan F., Zang Z., Xu Y., Zhang Q., Wang T., Huang X. (2024). Drying Characteristics of Moutan Cortex by Rotary Wheel Microwave Vacuum Drying and Its Influence on Quality. Agriculture.

[27] Huang Y., Chen Q., Pan W., Zhang Y., Li J., Xue X., Lei X., Wang S., Meng J. (2023). Moutan cortex exerts blood-activating and anti-inflammatory effects by regulating coagulation-inflammation cascades pathway in cells, rats and zebrafish. J. Ethnopharmacol..

[28] Cheng L., Ye L., Zhang Y., Wu M., Cao H., Ma Z. (2023). Study on drying characteristics and kinetics of Moutan Cortex decoction pieces based on LF-NMR/MRI and TA-HD plus. J. Jinan Univ..

[29] Huang X., Li W., Wang Y., Wan F. (2021). Drying characteristics and quality of Stevia rebaudiana leaves by far-infrared radiation. LWT-Food Sci. Technol..

[30] Shang J., Zhang Q., Wang T., Xu Y., Zang Z., Wang F., Yue Y., Huang X. (2023). Effect of Ultrasonic Pretreatment on the Far-Infrared Drying Process and Quality Characteristics of Licorice. Foods.

[31] Zhang Q., Wan F., Zang Z., Jiang C., Xu Y., Huang X. (2022). Effect of ultrasonic far-infrared synergistic drying on the characteristics and qualities of wolfberry (*Lycium barbarum* L.). Ultrason. Sonochem..

[32] National Pharmacopoeia Commission (2020). Chinese Pharmacopoeia.

[33] Ni J., Zielinska M., Wang J., Fang X., Sutar P.P., Li S., Li X., Wang H., Xiao H. (2023). Post-harvest ripening affects drying behavior, antioxidant capacity and flavor release of peach via alteration of cell wall polysaccharides content and nanostructures, water distribution and status. Food Res. Int..

[34] Yamchi A.A., Yeganeh R., Kouchakzadeh A. (2022). Effect of ultrasonic pretreatment on drying kinetics and physio-mechanical characteristics of peach slices. J. Food Process Eng..

[35] Chao E., Tian J., Fan L., Zhang T. (2021). Drying methods influence the physicochemical and functional properties of seed-used pumpkin. Food Chem..

[36] Yue Y., Zang Z., Wan F., Zhang Q., Shang J., Xu Y., Jiang C., Wang T., Huang X. (2022). Effect of Ultrasonic Pretreatment on Radio Frequency Vacuum Drying Characteristics and Quality of Codonopsis pilosula Slices. Agriculture..

[37] Feng Y., Tan C.P., Zhou C., Yagoub A.E.G.A., Xu B., Sun Y., Ma H., Xu X., Yu X. (2020). Effect of freeze-thaw cycles pretreatment on the vacuum freeze-drying process and physicochemical properties of the dried garlic slices. Food Chem..

[38] Lay M., Anuar S.A., Sadeh M., Nurestri A. (2014). Phytochemical constituents, nutritional values, phenolics, flavonols, flavonoids, antioxidant and cytotoxicity studies on phaleria macrocarpa (Scheff.) Boerl fruits. BMC Complement. Altern. Med..

[39] Xu Y., Xiao D., Lagnika C., Song J., Li D., Liu C., Jiang N., Zhang M., Duan X. (2020). A comparative study of drying methods on physical characteristics, nutritional properties and antioxidant capacity of broccoli. Dry. Technol..

[40] Wang H., Liu Z., Vidyarthi S.K., Wang Q., Gao L., Li B., Wei Q., Liu Y., Xiao H. (2020). Effects of different drying methods on drying kinetics, physicochemical properties, microstructure, and energy consumption of potato (*Solanum tuberosum* L.) cubes. Dry. Technol..

[41] Zhang J., Li M., Cheng J., Wang J., Ding Z., Yuan X., Zhou S., Liu X. (2019). Effects of Moisture, Temperature, and Salt Content on the Dielectric Properties of Pecan Kernels during Microwave and Radio Frequency Drying Processes. Foods.

[42] Jiang C., Wan F., Zang Z., Zhang Q., Xu Y., Huang X. (2022). Influence of far-infrared vacuum drying on drying kinetics and quality characteristics of Cistanche slices. J. Food Process. Preserv..

[43] Huang Z., Zhu H., Yan R., Wang S. (2015). Simulation and prediction of radio frequency heating in dry soybeans. Biosyst. Eng..

[44] Wang W., Tang J., Zhao Y. (2021). Investigation of hot-air assisted continuous radio frequency drying for improving drying efficiency and reducing shell cracks of inshell hazelnuts: The relationship between cracking level and nut quality. Food Bioprod. Process..

[45] Ji Z., Zhao D., Yin J., Ding S., Liu X., Hao J. (2024). Quality analysis and pectin characteristics of winter jujube processed by microwave coupled with pulsed vacuum drying (MPVD). LWT-Food Sci. Technol..

[46] Dinani S.T., Hamdami N., Shahedi M., Havet M. (2015). Quality assessment of mushroom slices dried by hot air combined with an electrohydrodynamic (EHD) drying system. Food Bioprod. Process..

[47] Zang Z., Huang X., Zhang Q., Jiang C., Wang T., Shang J., He C., Wan F. (2023). Evaluation of the effect of ultrasonic pretreatment on vacuum far-infrared drying characteristics and quality of Angelica sinensis based on entropy weight-coefficient of variation method. J. Food Sci..

[48] Deng L., Xiong C., Sutar P.P., Mujumdar A.S., Pei Y., Yang X., Ji X., Zhang Q., Xiao H. (2022). An emerging pretreatment technology for reducing postharvest loss of vegetables-a case study of red pepper (*Capsicum annuum* L.) drying. Dry. Technol..

[49] Zhang J., Zheng X., Xiao H., Shan C., Li Y., Yang T. (2024). Quality and Process Optimization of Infrared Combined Hot Air Drying of Yam Slices Based on BP Neural Network and Gray Wolf Algorithm. Foods.

[50] Zhao Y., Bi J., Yi J., Njoroge D., Peng J., Hou C. (2018). Comparison of dynamic water distribution and micro-structure formation of shiitake mushrooms during hot air and far infrared radiation drying by low-field nuclear magnetic resonance and scanning electron microscopy. J. Sci. Food Agric..

[51] Zhou X., Xu R., Zhang B., Pei S., Liu Q., Ramaswamy H.S., Wang S. (2018). Radio Frequency-Vacuum Drying of Kiwifruits: Kinetics, Uniformity, and Product Quality. Food Bioprocess Technol..

[52] Yao L., Fan L., Duan Z. (2020). Effect of different pretreatments followed by hot-air and far-infrared drying on the bioactive compounds, physicochemical property and microstructure of mango slices. Food Chem..

[53] Li W., Yang R., Xia Y., Shao X., Wang Y., Zhang W. (2024). Image recognition technology provides insights into relationships between anthocyanin degradation and color variation during jet drying of black carrot. Food Chem..

[54] Kahraman O., Malvandi A., Vargas L., Feng H. (2021). Drying characteristics and quality attributes of apple slices dried by a non-thermal ultrasonic contact drying method. Ultrason. Sonochem..

[55] Garrido I., Hernández M.S., Llerena J.L., Espinosa F. (2022). Effect of Water Supplementation on Oxidant/Antioxidant Activities and Total Phenol Content in Growing Olives of the Morisca and Manzanilla Varieties. Antioxidants.

[56] Salehi F., Inanloodoghouz M. (2023). Effects of gum-based coatings combined with ultrasonic pretreatment before drying on quality of sour cherries. Ultrason. Sonochem..

[57] Konstantinos P., Penta P., John B.G., Costas E.S., Michael C.B., Christopher J.S., Quan V.V. (2017). Effect of vacuum-drying, hot air-drying and freeze-drying on polyphenols and antioxidant capacity of lemon (*Citrus limon*) pomace aqueous extracts. Int. J. Food Sci. Technol..

[58] Peinado J., Lerma N.L., Moreno J.A., Peinado R.A. (2008). Antioxidant activity of different phenolics fractions isolated in must from Pedro Ximenez grapes at different stages of the off-vine drying process. Food Chem..

